# Infection of zebrafish embryos with live fluorescent *Streptococcus pneumoniae* as a real-time pneumococcal meningitis model

**DOI:** 10.1186/s12974-016-0655-y

**Published:** 2016-08-19

**Authors:** Kin Ki Jim, JooYeon Engelen-Lee, Astrid M. van der Sar, Wilbert Bitter, Matthijs C. Brouwer, Arie van der Ende, Jan-Willem Veening, Diederik van de Beek, Christina M. J. E. Vandenbroucke-Grauls

**Affiliations:** 1 Department of Medical Microbiology and Infection Control, VU University Medical Center, De Boelelaan 1108, 1081 HZ Amsterdam, The Netherlands; 2 Department of Neurology, Center of Infection and Immunity Amsterdam (CINIMA), Academic Medical Center, University of Amsterdam, Meibergdreef 9, 1105 AZ Amsterdam, The Netherlands; 3 Department of Medical Microbiology, Center of Infection and Immunity Amsterdam (CINIMA), Academic Medical Center, University of Amsterdam, Meibergdreef 9, 1105 AZ Amsterdam, The Netherlands; 4 The Netherlands Reference Laboratory for Bacterial Meningitis, Academic Medical Center, University of Amsterdam, Meibergdreef 9, 1105 AZ Amsterdam, The Netherlands; 5 Molecular Genetics Group, Groningen Biomolecular Sciences and Biotechnology Institute, Centre for Synthetic Biology, University of Groningen, Nijenborgh 7, 9747 AG Groningen, The Netherlands; 6 Department of Medical Microbiology and Infection Control, VU University Medical Center, P.O. Box 7057, 1007 MB Amsterdam, The Netherlands

**Keywords:** Pneumococcal meningitis, Pneumolysin, Live cell imaging, *Streptococcus pneumoniae*, Zebrafish, Host-microbe interaction

## Abstract

**Background:**

*Streptococcus pneumoniae* is one of the most important causes of bacterial meningitis, an infection where unfavourable outcome is driven by bacterial and host-derived toxins. In this study, we developed and characterized a pneumococcal meningitis model in zebrafish embryos that allows for real-time investigation of early host-microbe interaction.

**Methods:**

Zebrafish embryos were infected in the caudal vein or hindbrain ventricle with green fluorescent wild-type *S. pneumoniae* D39 or a pneumolysin-deficient mutant. The *kdrl:mCherry* transgenic zebrafish line was used to visualize the blood vessels, whereas phagocytic cells were visualized by staining with far red anti-L-plastin or in *mpx:GFP*/*mpeg1:mCherry* zebrafish, that have green fluorescent neutrophils and red fluorescent macrophages. Imaging was performed by fluorescence confocal and time-lapse microscopy.

**Results:**

After infection by caudal vein, we saw focal clogging of the pneumococci in the blood vessels and migration of bacteria through the blood-brain barrier into the subarachnoid space and brain tissue. Infection with pneumolysin-deficient *S. pneumoniae* in the hindbrain ventricle showed attenuated growth and migration through the brain as compared to the wild-type strain. Time-lapse and confocal imaging revealed that the initial innate immune response to *S. pneumoniae* in the subarachnoid space mainly consisted of neutrophils and that pneumolysin-mediated cytolytic activity caused a marked reduction of phagocytes.

**Conclusions:**

This new meningitis model permits detailed analysis and visualization of host-microbe interaction in pneumococcal meningitis in real time and is a very promising tool to further our insights in the pathogenesis of pneumococcal meningitis.

**Electronic supplementary material:**

The online version of this article (doi:10.1186/s12974-016-0655-y) contains supplementary material, which is available to authorized users.

## Background

*Streptococcus pneumoniae* is a major cause of serious infections such as sepsis, pneumonia and meningitis. Despite advances in pneumococcal vaccines and effective antimicrobial therapy, the disease burden of invasive pneumococcal disease remains high, especially in resource-poor countries [1, 2]. Pneumococcal meningitis is a severe form of bacterial meningitis in children and adults [2–5]. The mortality rate ranges from 16 to 37 % in developed countries and up to 51 % in resource-poor areas [1, 4]. Approximately 30 to 52 % of patients surviving pneumococcal meningitis have disabling long-term neurological sequelae, such as focal neurologic deficits and cognitive slowness [5–7].

Susceptibility to and severity of pneumococcal meningitis are determined by host as well as pathogen characteristics [8, 9]. Immune status and disruption of the natural barriers of the brain are well-recognized factors influencing host susceptibility [1]. In recent years, the host’s genetic make-up has been increasingly recognized to determine susceptibility, for instance, due to genetic variation in innate immune receptors (Toll-like receptor 4), Fc gamma (Fc-γ) receptors and complement system [1, 8]. Also, the make-up of the pathogen is important; pneumococci harbour an array of virulence factors [10, 11]. The most important of these is the polysaccharide capsule with over 90 distinct serotypes identified. Carriage rates and invasiveness differ for the different serotypes [12]. The capsule protects the bacteria from opsonophagocytosis and inhibits complement activation [13]. Other important virulence factors include the cytolytic toxin pneumolysin and several cell-surface proteins, such as pneumococcal surface protein A (PspA) [10, 11]. The relationship between the bacterium and the host drives pneumococcal genome variation; less than 50 % of pneumococcal genes is present in all strains (the core genome) exemplifying this genome variability [14]. Both the presence and absence of genetic regions but also single nucleotide variations in the core genome can increase the pathogen’s capacity to cause disease and influence disease severity [9, 15].

To study pneumococcal virulence, different experimental murine models have been developed [16–18]. Limitations of these murine models include ethical issues, high costs and time needed for experiments; these limitations render mice not suitable for large-scale screening [17]. The zebrafish (*Danio rerio*) has emerged as a powerful vertebrate model to study infectious diseases caused by human pathogens or their related animal pathogens [19–22]. Zebrafish are teleost fish with an innate and adaptive immune system similar to the human immune system [22–24]. The innate immune system is already active at very early stages during zebrafish embryo development, whereas the adaptive immune system is active 4–6 weeks post fertilization [23]. Another advantage of this model is the unique ability to study host-pathogen interaction in real time because of the transparency of zebrafish embryos and the wide range of available fluorescent tools [22, 25]. Other advantages include high fecundity of the zebrafish, external development of the embryo and availability of gene-editing tools and tools to manipulate gene expression [26]. Additionally, this model can be used for medium-throughput screening to identify bacterial mutants with altered virulence or medium-throughput screening of pharmacological compounds [27, 28]. Recently, this model has been adapted to visualize and study mycobacterial meningitis — a central nervous system infectious disease [29]. Moreover, it has been shown that zebrafish embryos as well as adult zebrafish are susceptible to pneumococcal infection and develop meningitis [30, 31].

The aim of our study was to develop a zebrafish embryo infection model of pneumococcal meningitis that allows for real-time analysis of the host-pathogen interaction. To this end, we infected zebrafish embryos with a highly green fluorescent strain of pneumococcus that is still fully virulent [32]. Visualization of the infection was further improved by using a transgenic fish line (*kdrl*:*mCherry)* that has red fluorescent blood vessels, in combination with fluorescent far red staining of phagocytic cells with fluorescently labelled anti-L-plastin. Phagocyte dynamics were studied in more detail in a double-labelled *mpx*:*GFP*/*mpeg1:mCherry* zebrafish line with green fluorescent neutrophils and red fluorescent macrophages.

## Methods

### Bacterial strains and growth conditions

*S. pneumoniae* serotype 2 D39 wild-type strain and a pneumolysin-deficient D39 mutant were used [33, 34]. All pneumococcal strains were grown overnight on Columbia agar plates supplemented with 5 % defibrinated sheep blood at 37 °C in a humidified atmosphere with 5 % CO_2_. Green fluorescent *S. pneumoniae* D39 mutant strains were generated by fusing superfolder green fluorescent protein (sfGFP) to the histone-like protein HlpA as described by Kjos et al. [32]. Pneumococcal strains labelled with HlpA-GFP are fully virulent and have been used for in vitro and in vivo imaging [32]. Transformants were selected on plates containing 4.5 μg/ml chloramphenicol. Bacteria were collected from an overnight culture and suspended in Todd Hewitt broth supplemented with 0.5 % yeast extract (Difco, Becton Dickinson) and grown to mid log phase at 37 °C. Cells were harvested by centrifugation (6000 rpm, 10 min), washed with sterile phosphate-buffered saline (PBS), suspended in PBS with 20 % glycerol to obtain the desired concentrations and stored at −80 °C. Before injection, bacteria were suspended in sterile 0.5 % (*w*/*v*) phenol red solution (Sigma-Aldrich; P0290) to aid visualization of the injection process. The number of colony-forming units (CFU) per injection was determined by quantitative plating of the injection volume.

### Zebrafish husbandry, embryo care and injection procedure

Adult *Tg(kdrl:mCherry)*^*s896*^ wild-type zebrafish expressing red fluorescence in the blood vessel endothelial cells, adult double-labelled *Tg(mpx:GFP)*^*i114*^*/Tg (mpeg1:mCherry)*^*gl23*^ expressing green neutrophils and red macrophages, and the transparent adult *casper* mutant zebrafish *(mitfa*^*w2/w2*^*;roy*^*a9/a9*^*)* were maintained at 26 °C in aerated 5-L tanks with a 10/14 h dark/light cycle [35–38]. Zebrafish embryos were collected within the first hours post fertilization (hpf) and kept at 28 °C in E3 medium (5.0 mM NaCl, 0.17 mM KCl, 0.33 mM CaCl·2H_2_O, 0.33 mM MgCl_2_·7H_2_O) supplemented with 0.3 mg/L methylene blue. Embryos collected from *Tg(kdrl:mcherry)*^*s896*^ wild-type zebrafish and *Tg(mpx:GFP)*^*i114*^*/Tg (mpeg1:mCherry)*^*gl23*^ wild-type zebrafish were additionally treated with 0.003 % (*v*/*v*) 1-phenyl 2-thiourea (PTU) to inhibit the formation of melanocytes [39]. Prior to injection and live imaging, 2 and 4 days post fertilization (dpf) embryos were mechanically dechorionated if necessary and anaesthetised in 0.02 % (*w*/*v*) buffered 3-aminobenzoic acid methyl ester (pH 7.0) (Tricaine; Sigma-Aldrich, A5040). The zebrafish embryos were individually infected by microinjection with 1 nl of *S. pneumoniae* either in the hindbrain ventricle or in the caudal vein as described elsewhere [40]. All procedures involving zebrafish embryos were according to local animal welfare regulations.

### Survival experiments in infected zebrafish embryos

After infection, *casper* mutant zebrafish embryos were kept in 6-well plates at 28 °C with 20 individually injected embryos in each group per well. The mortality rate was determined by monitoring live and dead embryos at fixed time points between 12 and 120 hours post injection (hpi). All experiments were performed in triplicates.

### Fluorescence imaging of zebrafish embryos

Screening and imaging of HlpA-GFP *S. pneumoniae-*infected and non-infected zebrafish embryos were performed with a Leica MZ16FA fluorescence microscope with a Leica DFC420C camera attached. Non-infected control zebrafish embryos and embryos with visible fluorescent bacteria after infection were selected at previously determined time points and fixated overnight in 4 % paraformaldehyde in PBS. Subsequently, the embryos were stored in 100 % methanol at −20 °C for maximal of 2 month or until further use. Confocal images were generated with a Leica TCS SP8 Confocal Microscope. For optimal imaging, embryos were embedded in 1.5 % low-melting-point agarose dissolved in PBS in an open uncoated 8-well microscopy μ-Slide (http://ibidi.com). Leica Application Suite X software was used to process the confocal images, specifically for brightness/contrast enhancements as well as for creating merged images.

### Time-lapse fluorescence imaging of zebrafish embryos

Time-lapse bright-field and fluorescence images were acquired with a Zeiss Axio Zoom V16 stereo microscope. Double-labelled *Tg(mpx:GFP)*^*i114*^*/Tg(mpeg1:mCherry)*^*gl23*^ zebrafish embryos were imaged 15 min after injection in the hindbrain ventricle with wild-type *S. pneumoniae*. Images were obtained at 1 min intervals for 2.5 h. Embryos were embedded in 1.5 % low-melting-point agarose dissolved in egg water (60 μg/mL sea salts (Sigma-Aldrich; S9883) in MiliQ) in a 35-mm Petri dish immediately after injection and kept at 28 °C using a custom-made temperature-controlled beaker with a glass bottom and heated lid to avoid condensation. Zeiss Zen Pro, Photoshop CS6 and ImageJ software were used to process the time-lapse images, specifically for brightness/contrast enhancements as well as for stitching images.

### Immunohistochemical staining

Phagocytic cells of zebrafish embryos were stained with an anti-L-plastin staining [41, 42]. The anti-L-plastin was a kind gift from P. Morgan (Bristol University, UK). Briefly, stored, frozen embryos were rehydrated and rinsed with PBTx (1 % Triton X-100 in PBS), permeated in 0.24 % trypsin in PBS and blocked for 3 h in block buffer (10 % normal goat serum (NGS) in 1 % PBTx) to minimize non-specific binding of the antibodies. Incubation with anti-L-plastin (1:500 *v*/*v* dilution) in antibody buffer (1 % (*v*/*v*) NGS and 1 % (*w*/*v*) bovine serum albumin (BSA) in 1 % PBTx was done overnight at room temperature on a seesaw rocker. After washing with PBTx and incubation for 1 h in the block buffer, embryos were incubated with Alexa 647 goat-anti-rabbit secondary antibody (Life technologies, 1:400 dilution) overnight at 4 °C.

### Histopathological analysis

For histopathological analysis, 2 and 4 dpf *casper* mutant zebrafish embryos were infected with wild-type *S. pneumoniae* D39 via caudal vein or hindbrain injection. The zebrafish embryos were anaesthetised with tricaine, fixated in 4 % paraformaldehyde in PBS, embedded in paraffin and sectioned sagittally in 4 μm thickness. The sections were mounted on StarFrost microscope slides, and Nissl staining was used. The stained slides were scanned with a Menari D-SIGHT *fluo* scanner (Florence, Italy) at ×100 magnification with oil immersion for histopathological evaluation.

### Graphs and statistical analysis

Statistics and graphs were generated with GraphPad Prism 6.0. Survival data were analysed with the log rank (Mantel-Cox) test. Results were considered statistically significant at *p* values of <0.05 %.

## Results

### Hindbrain ventricle and caudal vein injection with *S. pneumoniae* cause a fulminant, dose-dependent infection in zebrafish embryos

#### Hindbrain ventricle infection

**Fig. 1:**
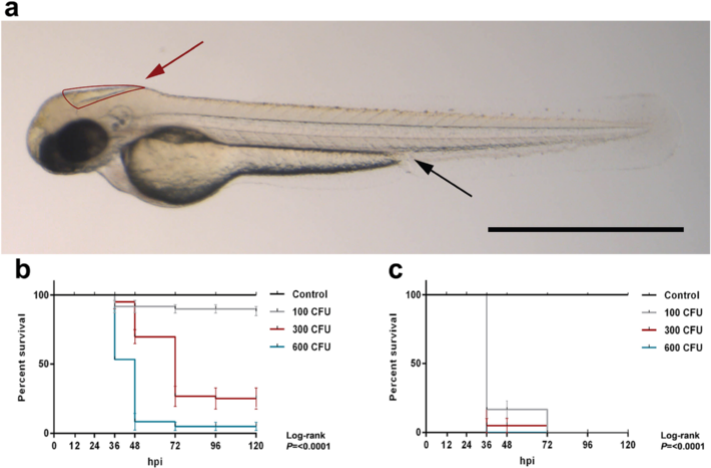
Survival curves of 2 days post-fertilization embryos injected through different routes with wild-type *Streptococcus pneumoniae* D39. **a** *Casper* zebrafish embryo at 2 days post - fertilization. *Red arrow* indicates the hindbrain ventricle infection route, and *black arrow* indicates the caudal vein infection route. Scale bar, 500 μm. **b**, **c** Injection in the **b** hindbrain ventricle (HBV) or **c** caudal vein with indicated doses. *Hpi* hours post injection, *CFU* colony-forming units. The data represent the mean ± SEM of three individual experiments with 20 embryos in each group

**Fig. 2:**
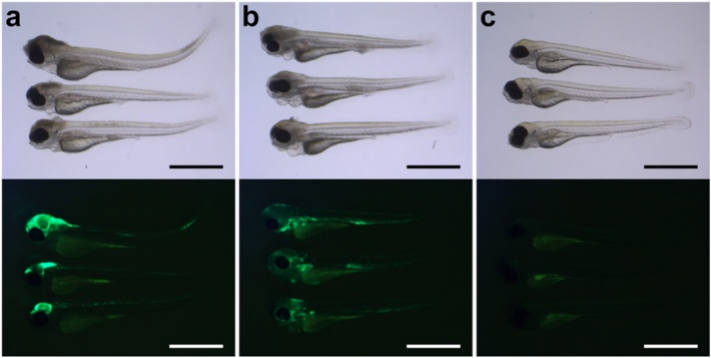
Bright-field images with corresponding fluorescent images of 2 day-post-fertilization zebrafish embryos infected by different routes. **a**, **b** *Lateral view* of zebrafish embryos injected in the **a** hindbrain ventricle or **b** in the caudal vein. **c** Non-injected control embryos. Please note that there is usually some background fluorescence observed in the yolk. All embryos were infected with 400 CFU of *Streptococcus pneumoniae* D39 (HlpA-GFP) and imaged at 48 h post injection. Scale bars, 500 μm

To study whether *S. pneumoniae* can cause meningitis in zebrafish embryos, we directly injected bacteria in the hindbrain ventricle (Fig. 1a; red arrow). Infection was dose-dependent in 2 dpf embryos: higher doses of bacteria resulted in earlier onset of disease and higher mortality rate. The survival of combined experiments at 120 hpi was 52 of 60 (88 %) embryos injected with 100 CFU, 15 of 60 (25 %) embryos injected with 300 and 3 of 60 (5 %) embryos injected with 600 CFU (Fig. 2b).

**Fig. 3:**
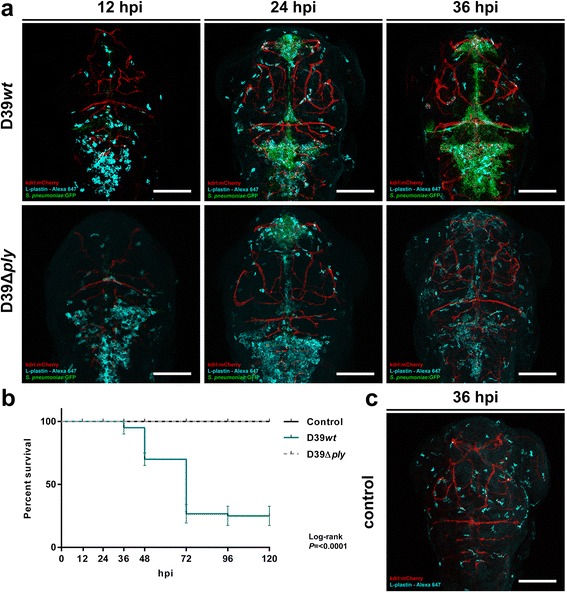
Comparison of 2 days post-fertilization zebrafish embryos infected with wild-type *Streptococcus pneumoniae* D39 (D39*wt*) or pneumolysin-deficient mutant strain (D39Δ*ply*). **a** Confocal microscopy images at maximum projection of *Tg(kdrl:mcherry)* ^*s896*^ zebrafish embryos infected with D39*wt* or D39Δ*ply* at different time points. **c** Non-infected zebrafish embryos. D39*wt* pneumococci grow rapidly compared to D39Δ*ply* and migrate throughout the subarachnoid space, delineating the ventricular contours. The numbers of phagocytes reduce over time in the presence of increasing numbers of D39*wt* bacteria compared to non-infected or D39Δ*ply* zebrafish embryos. Embryos were infected with 600 CFU. Scale bars, 100 μm. **b** Corresponding survival curves. Embryos were infected with 300 CFU. The data represent three individual experiments with 20 embryos in each group

**Fig. 4:**
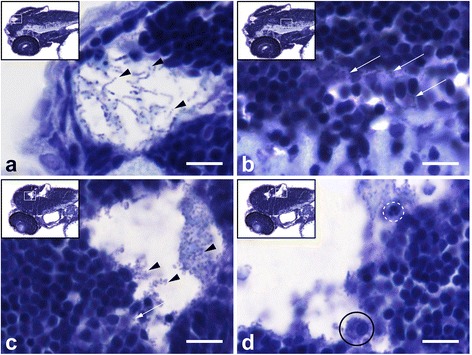
Histopathological analysis of *Streptococcus pneumoniae*-infected zebrafish embryos via the hindbrain ventricle at 2 days post fertilization. **a**, **b** Sagittal section of the head region showing bacteria (*arrow heads*) in **a** the subarachnoid space and **b** brain parenchyma at 12 h post injection (hpi). **c**, **d** Sagittal section at 24 hpi showing increased amount of bacteria in the subarachnoid space (*arrow heads*) and disruption of the ventricular lining with bacterial infiltration (*arrow*) in **c** and a neutrophil (*dotted circle*) and a phagocytosing macrophage (*circle*) in **d**. Scale bars, 10 μm

To visualize the localization of bacterial infiltrates, we used the green fluorescent HlpA-GFP *S. pneumoniae* D39 strain. After injection in the hindbrain ventricle, the infection remained mainly confined to the central nervous system (Fig. 2a). When smaller infection doses were used (~300 CFU), death was delayed and sometimes preceded by bacteraemia (Additional file 1: Figure S1). To study the dynamics between *S. pneumoniae* and the innate immune system in more detail, phagocytes of infected zebrafish embryos were stained with anti-L-plastin and followed over time. Pneumococci injected in the hindbrain ventricle grew rapidly in number and migrated throughout the subarachnoid space, delineating the ventricular contours. In the early phase of infection, phagocytes migrated in large numbers to the site of infection. As the infection continued to progress, the number of phagocytes in the hindbrain reduced over time in the presence of increasing numbers of bacteria (Fig. 3a), possibly due to the cytotoxic activity of pneumolysin. Histopathological analysis showed a similar pattern of increasing amount of bacteria in the subarachnoid space over time. In addition, bacteria were able to infiltrate the brain parenchyma (Fig. 4).

#### Systemic infection

**Fig. 5:**
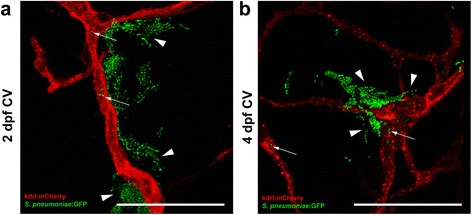
Pneumococci leave the blood vessels after systemic infection. **a**, **b** Confocal microscopy images at maximum projection of *Tg(kdrl:mcherry)* ^*s896*^ zebrafish embryos injected in the caudal vein (CV) **a** before formation of the blood-brain barrier (BBB) at 2 days post fertilization (dpf) or **b** after the formation of the BBB at 4 dpf. Bacteria were localized inside (*arrows*) and outside (*arrow heads*) the blood vessels. All embryos were infected with 400 CFU and imaged at 24 h post injection. Scale bars, 50 μm

**Fig. 6:**
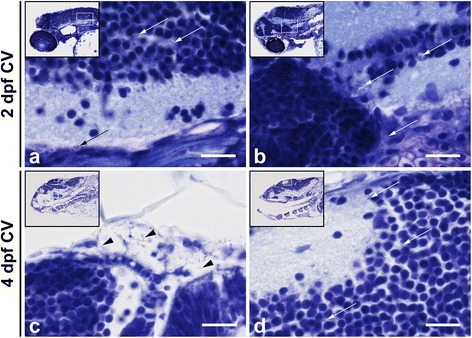
Histopathological analysis of *Streptococcus pneumoniae*-infected zebrafish embryos via the caudal vein at 2 days post - fertilization. **a**, **b** Caudal vein injection at 2 days post - fertilization (dpf). Sagittal section of the head region showing the bacteria (*arrows*) in the brain parenchyma at **a** 12 h post injection (hpi) and at **b** 24 hpi. **c**, **d** Caudal vein-injected zebrafish embryos at 4 dpf. **c** Sagittal section at 24 hpi showing bacteria (*arrows*) in the meningeal space and in **d** the brain parenchyma. Scale bars, 10 μm

We injected 2 dpf zebrafish embryos with HlpA-GFP *S. pneumoniae* in the caudal vein (Fig. 1a; black arrow). Injection of increasing doses of pneumococci in the caudal vein resulted in a dose-dependent infection. The mean survival percentages of the combined experiments were 0 % in 100 CFU/embryo, 300 CFU/embryo and 600 CFU/embryo (Fig. 1c), clearly indicating that compared to hindbrain ventricle injection, injection in the caudal vein was associated with a more rapid disease progression (Fig. 1b, c). Bacterial infiltrates were observed throughout the whole body of the zebrafish and also in the central nervous system (Fig. 2b). Injection in the caudal vein of *Tg(kdrl:mCherry)*^*s896*^ zebrafish embryos allowed for detailed examination of the relation between the bacteria and the cerebrovascular system and showed pneumococci migrating out of the blood vessels into the central nervous system (Fig. 5). Histopathological analysis showed bacteria in the brain parenchyma as early as 12 hpi (Fig. 6).

#### Pneumolysin-deficient mutant is attenuated in the zebrafish embryo meningitis model

To study whether a pneumolysin-deficient mutant pneumococcal strain (D39Δ*ply*) was attenuated as compared to the wild-type strain in our zebrafish embryo meningitis model, we injected the mutant bacteria in the hindbrain ventricle with equal doses as the wild-type bacteria (Fig. 1a). Infection with *S. pneumoniae* D39Δ*ply* in the hindbrain ventricle showed attenuated growth and migration through the subarachnoid space and brain as compared to the wild-type D39 strain (Fig. 3a). The mean survival percentage 5 dpi was significantly higher compared to zebrafish embryos infected with the wild-type strain (100 vs 25 %, *P* = <0.0001) (Fig. 3b). To visualize the differences in infection dynamics between the pneumolysin-deficient mutant and the wild-type strain, *Tg(kdrl:mCherry)*^*s896*^, zebrafish embryos were infected with the green fluorescent HlpA-GFP *S. pneumoniae* D39Δ*ply* strain and phagocytes were stained with anti-L-plastin. At early stages of infection, bacteria were observed in the subarachnoid space with large numbers of phagocytes at the site of infection. The number of bacteria reduced over time in the presence of increasing numbers of phagocytes (predominantly macrophages) (Fig. 3a and Additional file 2: Figure S2). At 36 hpi, no bacteria were found in the subarachnoid space, while phagocytes continued to delineate its contours. This pattern was distinct from that in non-infected zebrafish embryos, where phagocytes appeared scattered throughout the whole brain at this time point (Fig. 3c). These findings were consistent with the survival data, showing attenuated virulence of *S. pneumoniae* D39Δ*ply* compared to wild-type *S. pneumoniae* D39 and also seem to confirm that reduction of phagocytes in wild-type infections at later time points is probably due to pneumolytic activity.

### Infection before and after formation of the blood-brain barrier

**Fig. 7:**
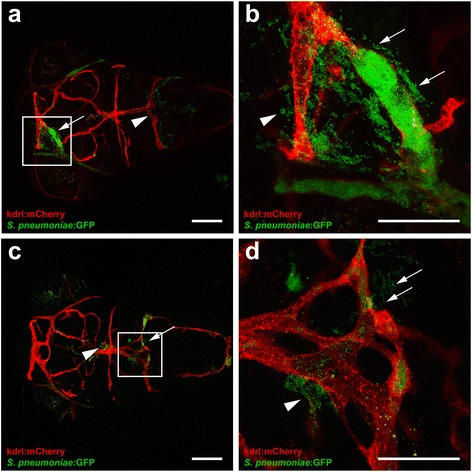
Clogging of the blood vessels by *Streptococcus pneumoniae* after systemic infection. **a**–**d** Confocal microscopy images at maximum projection of *Tg(kdrl:mcherry)* ^*s896*^ zebrafish embryos at 4 days post- fertilization injected in the caudal vein. **a**, **c** Bacteria were localized inside and outside of the blood vessels with (*arrows*) and without clogging (*arrow heads*). Scale bars, 100 μm. **b**, **d** An enlarged view of **a** and **c**, respectively, with clogging of a blood vessel highlighted. All embryos were infected with 600 CFU and imaged at 24 h post injection. Scale bars, 50 μm

The blood-brain barrier (BBB) forms on day 3 post - fertilization and continues to mature till 10 dpf in zebrafish [43–45]. To examine whether the blood-brain barrier influences the migration of bacteria to the central nervous system, we injected HlpA-GFP wild-type *S. pneumoniae* D39 in the caudal vein of 4 dpf *Tg(kdrl:mCherry)*^*s896*^ zebrafish embryos (after formation of BBB) and compared this with 2 dpf infected zebrafish embryos (before formation of BBB). In addition, we performed histopathological analysis with Nissl staining to determine the spreading and localization of bacteria in both 2 and 4 dpf zebrafish embryos. After bloodstream injection, pneumococci were seen migrating out of the blood vessels and into the central nervous system after 24 hpi in both 2  and 4 dpf zebrafish embryos (Fig. 5a, b). However, histopathological analysis showed bacterial infiltration of brain parenchyma at 12 hpi in 2 dpf zebrafish embryos and at 24 hpi in 4 dpf zebrafish embryos (Fig. 6). Interestingly, clogging of the blood vessels by pneumococci was commonly observed in caudal vein-infected zebrafish embryos and bacteria were frequently found outside of these vessels (Fig. 7). Together, these data indicate that migration to the subarachnoid space and brain tissue occurs in zebrafish before and after the formation of the blood-brain barrier, be it that after the formation of the blood-brain barrier this migration occurs at a later time point.

### Initial innate immune response during pneumococcal meningitis in zebrafish embryos consists mainly of neutrophils

**Fig. 8:**
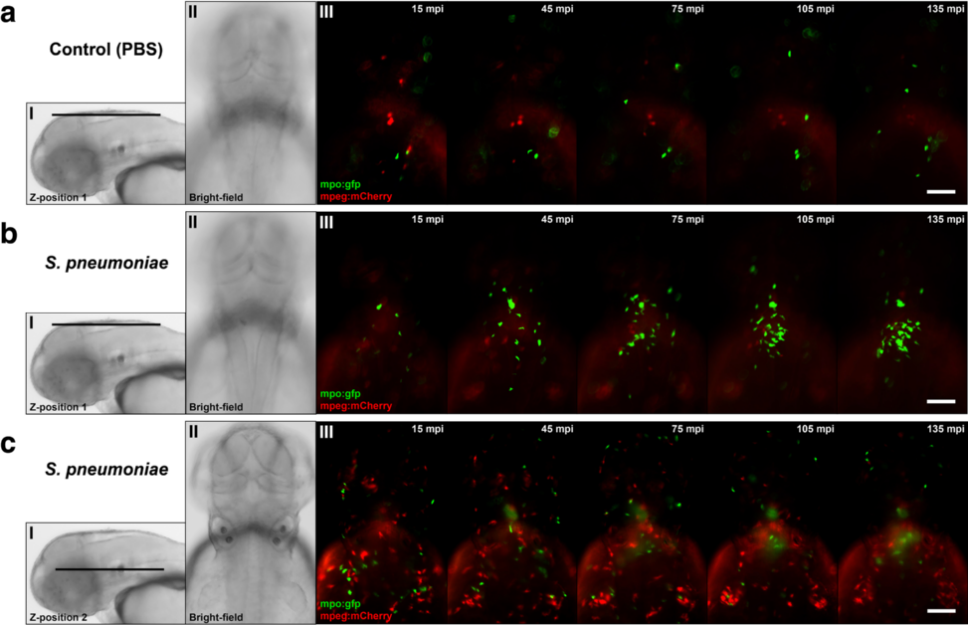
Time-lapse fluorescence imaging of *Streptococcus pneumoniae-*infected zebrafish embryos via the hindbrain ventricle at 2 days post fertilization. **a**–**c** Live fluorescence images of double-labelled *Tg(mpx:GFP)* ^*i114*^ */Tg (mpeg1:mCherry)* ^*gl23*^ zebrafish embryos (green fluorescent neutrophils, red fluorescent macrophages). Dorsal view of the head region after injection with **a** PBS or **b**, **c** *S. pneumoniae* D39 with the corresponding *Z*-position (*I*) and bright-field image (*II*). Images **b** and **c** were acquired from the same zebrafish embryo at different positions. **b** (*III*) After injection of pneumococci in the hindbrain ventricle, green fluorescent neutrophils migrate in increasing numbers to the site of infection compared to **a** (*III*) PBS-injected zebrafish embryos. Macrophages were not observed in the subarachnoid space during **b** early pneumococcal infection of the hindbrain ventricle but **c** (*III*) remain localized in regions below the subarachnoid space. Scale bars, 100 μm

To study the dynamics of the innate immune response against *S. pneumoniae*, we injected *Tg(mpx:GFP)*^*i114*^*/Tg (mpeg1:mCherry)*^*gl23*^ zebrafish embryos in the hindbrain ventricle with wild-type *S. pneumoniae* and performed time-lapse fluorescence imaging (Additional file 3: Movie S1). From as early as 15 min after injection, neutrophils migrated into the subarachnoid space. While neutrophils accumulated over time, no macrophages appeared to move towards the infection site during this early stage of infection, despite their presence in regions below the subarachnoid space (Fig. 8b, c). Zebrafish embryos injected with PBS showed no migration of neutrophils or macrophages (Fig. 8a). This shows that neutrophils are the primary immune cells that are migrating towards loci of pneumococcal infection in the brain tissue.

## Discussion

We developed and characterized a zebrafish embryo infection model of pneumococcal meningitis allowing real-time investigation of early host-microbe interaction. Meningitis developed both after systemic injection in the caudal vein or local injection in the hindbrain ventricle. Infection with a pneumolysin-deficient pneumococcal mutant strain in the hindbrain ventricle showed attenuated growth in the subarachnoid space and attenuated migration through the brain as compared to the wild-type strain. In the wild-type strain infection, the number of phagocytes reduced quickly after initial accumulation at the site of infection, in contrast to the pneumolysin-deficient mutant infection, where numbers of phagocytic cells kept accumulating in the subarachnoid space. This observation suggested that cytolytic activity, mediated by pneumolysin, may be responsible for the reduction of phagocytic cells. Time-lapse imaging showed that the initial zebrafish phagocytic innate immune response in pneumococcal meningitis mainly consisted of neutrophils, comparable to the human situation [4].

Zebrafish embryos and larvae are becoming increasingly popular to model infectious diseases, including infections of the central nervous system [19, 21, 26, 29, 46, 47]. The optical clarity of zebrafish embryos and larvae in conjunction with transgenic zebrafish lines, fluorescently labelled bacteria and immunohistochemistry provide unique possibilities for real-time in vivo imaging of infection dynamics in the central nervous system in detail. This approach has led to the successful modelling of tuberculous meningitis and *Streptococcus agalactiae* meningitis in zebrafish embryos and better understanding of the molecular and cellular pathogenesis of the disease [29, 46]. Given the potential of this model to study central nervous system infections, it is therefore recommended and desirable to further adapt the zebrafish embryo to study other forms of bacterial meningitis. Since it has been demonstrated that zebrafish are susceptible to *Listeria monocytogenes*, *Streptococcus suis*, *Streptococcus iniae* and *Escherichia coli* infection, zebrafish meningitis models should be developed for these bacteria and opportunities for other meningitis-causing pathogens explored [48–51].

Injection of wild-type *S. pneumoniae* in the hindbrain ventricle or caudal vein caused a fulminant dose-dependent infection in zebrafish embryos. Caudal vein injection was associated with more severe disease outcome as compared to hindbrain ventricle injection, suggesting tissue-specific susceptibility to pneumococcal infection. A similar trend was observed in *Staphylococcus aureus*-infected zebrafish embryos, where infection of the hindbrain ventricle elicited a stronger immunological response as compared to systemic infection [47]. A recent study showed that the innate immune response to pneumococcal infection in zebrafish embryos is highly dependent on phagocytic cells (macrophages and neutrophils) [30]. The difference in phagocyte recruitment upon injection via different routes may explain the difference in survival that we observed. The association between lack of leukocyte response and adverse outcome has been described before: a low cerebrospinal fluid white-cell count was associated with an adverse outcome in patients with pneumococcal meningitis [5]. Studies in rats showed a relation between large numbers of bacteria in the cerebrospinal fluid load, lack of response of cerebrospinal fluid leukocytes and intracranial complications [52].

Pneumolysin is a crucial multifunctional virulence factor, which is best known for its cholesterol-dependent cytolytic activity of host cells, but also induces activation of the complement pathway, activation of pro-inflammatory immune cell reactions and induction of apoptosis [53]. The role of pneumolysin in the pathogenesis of pneumococcal meningitis has been controversial [54]. Recent studies, however, show that pneumolysin plays an important role in the pathogenesis of pneumococcal meningitis and that unfavourable outcome in meningitis is driven by a combination of bacteria and host-derived toxins [4, 54–57]. In line with these studies, the pneumolysin-deficient mutant was attenuated as compared to the wild-type strain after hindbrain ventricle injection; real-time imaging showed the differences in innate immune response upon infection with these strains. After infection of the hindbrain ventricle with wild-type pneumococci, large numbers of phagocytes migrated to the site of infection. However, as the infection progressed, the numbers of phagocytes diminished over time in the presence of increasing numbers of bacteria. In contrast, after hindbrain ventricle infection with pneumolysin-deficient pneumococci, phagocytes remained present in large numbers while bacteria were cleared over time. These observations may be explained by a biological phenomenon called apoptosis-associated killing of bacteria. Macrophage apoptosis has been described as a mechanism for pneumococcal clearance when other killing mechanisms are exhausted and is initiated by lysosomal membrane permeabilization [58–60]. Induction of this mechanism by pneumococci requires opsonization and is correlated with the intracellular bacterial burden [59–61]. A recent study shows that pneumolysin is necessary for lysosomal membrane permeabilization and thus induction of macrophage apoptosis-associated killing of pneumococci [62]. In addition, in vitro studies show that infection using pneumolysin-deficient strains resulted in a significant reduction of macrophage apoptosis [59, 63]. Altogether, these data strongly suggest that the observed differences in host innate immune response between infection with wild-type and pneumolysin-deficient pneumococci may be due to the ability and necessity to activate this macrophage apoptosis-associated killing mechanism.

Bacterial meningitis develops when bacteria enter and survive in the bloodstream, interact with the BBB and penetrate the central nervous system [4]. In pneumococcal meningitis, crossing of the BBB by *S. pneumoniae* is thought to occur by intracellular or intercellular translocation, although the exact mechanisms remain unclear [4]. The BBB is formed by endothelial cells with tight junctions, astrocytes and pericytes, and the main function is to protect the central nervous system from microorganisms and toxins that are circulating in the blood [64]. Previous studies showed that zebrafish have a functional BBB similar to that of mammals, and are therefore suitable for studying mechanisms involved in the disruption and penetration of the BBB [43–45]. A recent study by Kim et al. demonstrated that infection with *S. agalactiae* in zebrafish induces the Snail1 host transcription factor, which downregulates tight junctions, and disrupts the BBB [65]. In order to investigate whether the zebrafish embryo model can be used to study pneumococcal crossing of the BBB, we infected *Tg(kdrl:mCherry)*^s896^ zebrafish embryos that express red fluorescence in the blood vessels with green fluorescent pneumococci. Wild-type pneumococci injected in the caudal vein migrated out of the blood vessels and caused meningitis in zebrafish embryos before as well as after the formation of the BBB. Histopathological analysis confirmed these findings and showed bacteria in the subarachnoid space and brain parenchyma in both 2 and 4 dpf zebrafish embryos infected systemically. This is in line with data from adult zebrafish, where intraperitoneal injection of pneumococci causes bacteraemia and subsequent meningitis [31]. These findings suggest that the zebrafish embryo model is suitable to elucidate the mechanism by which pneumococci cross the BBB in meningitis.

In our analysis, we also detected clogging of the blood vessels by pneumococci in the bloodstream-infected zebrafish embryos, with bacteria localized outside and in proximity to these affected vessels. In addition to the aforementioned mechanisms by which pneumococci can infiltrate the central nervous system, mechanical disruption of vascular endothelium by pneumococci could possibly be another mechanism by which pneumococci leave the bloodstream and invade the brain. Furthermore, clogging of the blood vessels may cause interruption of the blood flow and subsequent cerebral infarction in zebrafish embryos. In patients with pneumococcal meningitis, cerebral infarction has been described as a common complication [66–70]. Whereas the exact mechanism remains to be elucidated, previous studies show that severe infection can activate the coagulation pathway and diffuse intravasal coagulation may contribute to the pathogenesis of cerebral infarction [69, 71].

Although there are many advantages of the zebrafish as an infection model, there are also some limitations. First, most human pathogens are adapted to cause infection at 37 °C, whereas the ideal temperature for zebrafish is around 28 °C. The difference in temperature might influence the natural disease course of human pathogens in these animals. Translation from the zebrafish model to the human infectious disease might therefore not always be possible. Second, monoclonal antibodies directed to surface antigens of cells of the zebrafish immune system are scarce [21, 72]. Finally, the immune cells of the adaptive immune response that have been assumed to play a role in the innate immune response to pneumococcal infection show a different pattern in zebrafish as compared to mice and human [31, 73]. Moreover, there is evidence that zebrafish have a tissue-restricted expression of Toll-like receptors and the repertoire of components of the zebrafish innate immune system seems to be more diverse that in mice or humans [74, 75]. Despite these differences, the zebrafish embryo model has been proven very useful to study several human pathogens, e.g. *Mycobacterium tuberculosis*, and has provided important new insights in the pathogenesis of tuberculosis [76]. Also, our findings with respect to the pathogenesis of pneumococcal meningitis appear in line with those found in other animal models. Therefore, the zebrafish remains a powerful model organism to study infectious diseases.

## Conclusions

In conclusion, we have developed and characterized a novel zebrafish embryo infection model to visualize and study pneumococcal meningitis infection dynamics in detail. This model shows the potential to extend our understanding of the interplay between bacterial virulence factors and host defence mechanisms in the pathogenesis of pneumococcal meningitis. In addition, our observations stress the need for targeting direct bacterial toxicity, for example, targeting pneumolysin, to prevent host-derived toxin-mediated brain damage and associated poor disease outcome in pneumococcal meningitis.

## Abbreviations

BBB, blood-brain barrier; BSA, bovine serum albumin; CFU, colony-forming units; Fc-γ, fc gamma; GFP, green fluorescent protein; HlpA, histone-like protein; hpf, hours post fertilization; hpi, hours post injection; kdrl, kinase insert domain receptor like; mpeg, macrophage expressed gene; mpx, myeloperoxidase; NGS, normal goat serum; PBS, phosphate-buffered saline; PBTx, Triton X-100 in phosphate-buffered saline; ply, pneumolysin; PspA, pneumococcal surface protein A; PTU, 1-phenyl 2-thiourea; sfGFP, superfolder green fluorescent protein; Tg, transgenic

## Additional files

Additional file 1: Figure S1. Infection with green fluorescent wild-type HlpA-GFP *Streptococcus pneumoniae* D39 via the hindbrain ventricle can lead to systemic infection in zebrafish embryos. Fluorescence microscopy image at 36 h post injection. After infection of the embryos via the hindbrain ventricle, bacteria can disseminate into the bloodstream and cause a systemic infection. The embryo was infected at 2 days post-fertilization with 300 CFU of *Streptococcus pneumoniae* D39 (HlpA-GFP). Scale bar, 500 μm. (JPG 505 kb) Additional file 2: Figure S2. Macrophages are involved in the clearance of pneumolysin-deficient pneumococci (D39Δ*ply*) in zebrafish embryos after infection via the hindbrain ventricle. Confocal microscopy images at maximum projection of double-labelled *Tg(mpx:GFP)* ^*i114*^ */Tg (mpeg1:mCherry)* ^*gl23*^ zebrafish embryo (green fluorescent neutrophils (arrows), red fluorescent macrophages (*arrowheads*)) infected with 600 CFU *Streptococcus pneumoniae* D39Δ*ply* in the hindbrain ventricle at 2 days post fertilization and imaged at 24 h post injection. Scale bar, 50 μm. (JPG 902 kb) Additional file 3: Movie S1. Time-lapse movie showing the initial innate immune response of *Streptococcus pneumoniae*-infected zebrafish embryos in the hindbrain ventricle. Dorsal view of *Streptococcus pneumoniae-*infected double-labelled *Tg(mpx:GFP)* ^*i114*^ */Tg (mpeg1:mCherry)* ^*gl23*^ zebrafish embryo (green fluorescent neutrophils, red fluorescent macrophages) via the hindbrain ventricle at 2 days post fertilization. Fifteen minutes after the injection of pneumococci in the hindbrain ventricle, green fluorescent neutrophils migrate in increasing numbers to the site of infection, whereas macrophages were not observed in the hindbrain ventricle. Interval time between frames is 1 min for 2.5 h. Frame rate is 7 frames per second. (MP4 18374 kb)
