# Supplementary figures and images for: Infection of zebrafish embryos with live fluorescent Streptococcus pneumoniae as a real-time pneumococcal meningitis model
Source: J Neuroinflammation. 2016 Aug 19;13:188. doi: 10.1186/s12974-016-0655-y (PMC4992281; doi:10.1186/s12974-016-0655-y)

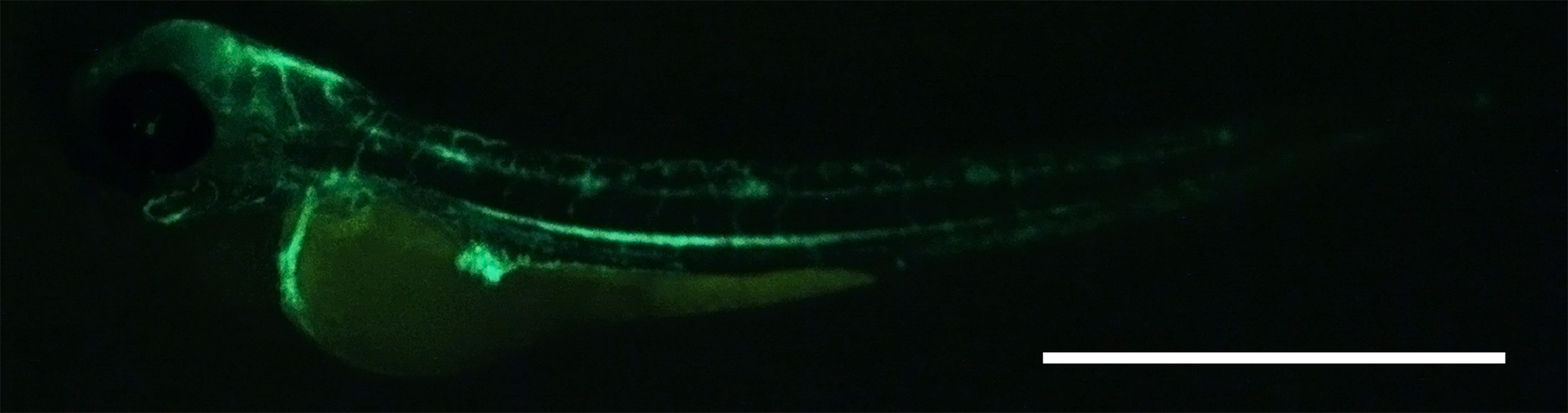

Supplement: Additional file 1: Figure S1. — Infection with green fluorescent wild-type HlpA-GFP Streptococcus pneumoniae D39 via the hindbrain ventricle can lead to systemic infection in zebrafish embryos. Fluorescence microscopy image at 36 h post injection. After infection of the embryos via the hindbrain ventricle, bacteria can disseminate into the bloodstream and cause a systemic infection. The embryo was infected at 2 days post-fertilization with 300 CFU of Streptococcus pneumoniae D39 (HlpA-GFP). Scale bar, 500 μm. (JPG 505 kb) [file 12974_2016_655_MOESM1_ESM.jpg]

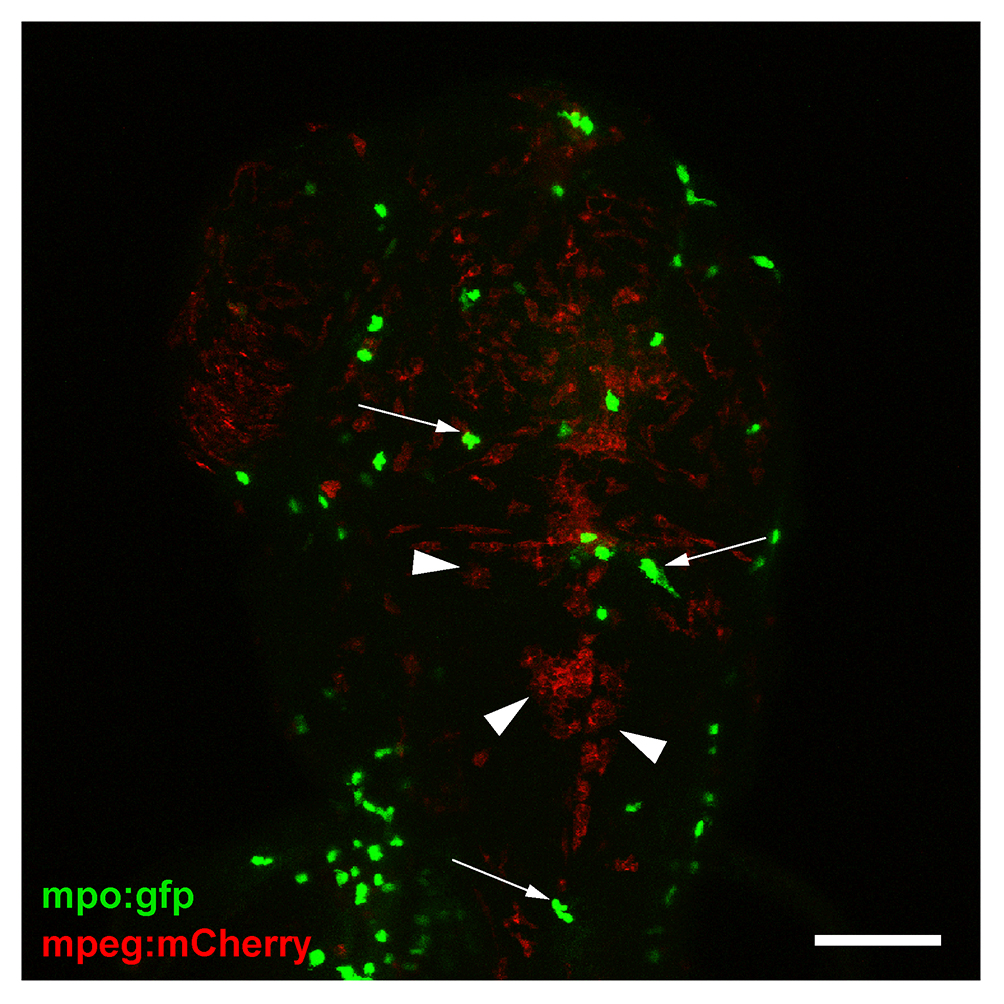

Supplement: Additional file 2: Figure S2. — Macrophages are involved in the clearance of pneumolysin-deficient pneumococci (D39Δply) in zebrafish embryos after infection via the hindbrain ventricle. Confocal microscopy images at maximum projection of double-labelled Tg(mpx:GFP) i114 /Tg (mpeg1:mCherry) gl23 zebrafish embryo (green fluorescent neutrophils (arrows), red fluorescent macrophages (arrowheads)) infected with 600 CFU Streptococcus pneumoniae D39Δply in the hindbrain ventricle at 2 days post fertilization and imaged at 24 h post injection. Scale bar, 50 μm. (JPG 902 kb) [file 12974_2016_655_MOESM2_ESM.jpg]
